# Differential MicroRNA Expression Profile between Stimulated PBMCs from HIV-1 Infected Elite Controllers and Viremic Progressors

**DOI:** 10.1371/journal.pone.0106360

**Published:** 2014-09-16

**Authors:** Lander Egaña-Gorroño, Tuixent Escribà, Nicolas Boulanger, Alberto Crespo Guardo, Agathe León, Manel Enric Bargalló, Felipe Garcia, José María Gatell, Montserrat Plana, Mireia Arnedo, HIV Controllers Consortium of the AIDS Spanish Network

**Affiliations:** 1 Group of Genomics and Pharmacogenomics, AIDS Research Group, Catalan project for the development of an HIV vaccine (HIVACAT), Institut d'Investigacions Biomèdiques August Pi i Sunyer (IDIBAPS), Hospital Clínic de Barcelona, Barcelona, Spain; 2 Immunopathology and Cellular Immunology, AIDS Research Group, Catalan project for the development of an HIV vaccine (HIVACAT), Institut d'Investigacions Biomèdiques August Pi i Sunyer (IDIBAPS), Hospital Clínic de Barcelona, Barcelona, Spain; 3 Department of Infectious Diseases, Hospital Clínic de Barcelona, University of Barcelona, Barcelona, Spain; 4 Centro Nacional de Análisis Genómico, Scientific Park of Barcelona, Barcelona, Spain; French National Center for Scientific Research - Institut de biologie moléculaire et cellulaire, France

## Abstract

**Background:**

The emerging relationship between microRNAs (miRNA) and viral-control is a topic of interest in the field of HIV. Host-genome might play an important role in the control of viremia. The aim of this study was to assess the specific miRNA profile that could contribute to the control of HIV replication in Elite Controllers

**Results:**

After adequate normalization, expression profile of 286 human miRNAs (hsa-miR) was evaluated in phytohaemagglutinin-stimulated PBMCs from 29 individuals classified in 4 groups: 8 elite controllers (EC; viral load <50 cp/ml without treatment), 8 viremic progressors (VP; VL>5000 cp/ml without treatment), 8 patients under antiretroviral treatment (ART; VL<200 cp/ml) and 5 uninfected individuals (HIV-) through TaqMan Array Human microRNA Cards v3.0. A differential expression pattern consisting of 23 miRNAs became significantly different when comparing EC and VP. Profiling analysis segregated the population in two different blocks: while EC and HIV- clustered together in the same block (EC/HIV-_block 1), VP and ART individuals clustered together in a second block (VP/ART_block 2). Two inversely expressed miRNA patterns were determined within those two blocks: a set of 4 miRNAs (hsa-miR-221, -27a, -27b and -29b) was up-expressed in EC/HIV-_block and down-expressed in VP/ART_block while 19 miRNAs were down-expressed in block 1 and up-expressed in block 2. Differential miRNAs were successfully validated through individual RT-qPCR assays.

**Conclusions:**

Profile in EC resembled HIV- and differentially clusters with VP and ART. Therefore, differential clustering does not rely on undetectable viremia.

## Introduction

The control of human immunodeficiency virus (HIV) replication is an intrinsic feature present in a subset of infected individuals known as Elite Controllers (EC). Contrary to viremic progressors (VP), who register high levels of viral load and exhibit a dramatic loss of CD4+ T-cells, more than 60% of EC have the ability to maintain high T-cell-counts and undetectable viral load (HIV RNA <50 copies/ml) in the absence of antiretroviral therapy (ART) [1]–[3]. The mechanisms associated with this extreme control of the viremia remains elusive [4]. However, the presence of a low viral reservoir or the existence of a potent CD8+ T-cell response, mainly against the structural protein *gag*, could partially explain this control [5].

There has been an effort to identify molecular, immunological and virological mechanisms leading to the susceptibility of HIV-1 infection, control of viral replication, and disease progression [6]–[8]. Genetically, EC have been shown to describe a composite of *CCR5* delta-32 gene deletion and/or certain class-I HLA alleles, such as HLA-B*57, that discriminate them from progressors [9]–[11]. However to date, there has been no clear explanation to why some subjects can control viremia in the absence of antiretroviral treatment and others cannot, even when carrying the same protective alleles. In addition, genome-wide associations studies and transcriptome analyses have been performed aiming to determine specific DNA variants and gene expression patterns present in HIV controllers [12]–[17]. Furthermore, the discovery of a growing class of small RNAs, termed microRNAs (miRNAs), has opened a new field of research and revealed the possibility to identify plausible miRNA profiles in the context of diseases, including HIV/AIDS and vaccines.

miRNAs are approximately 19–25 nucleotide long single-strand noncoding RNAs capable of regulating gene expression at the post-transcriptional level [18]–[20]. They pair to the messages of protein-coding genes, usually through imperfect base-pairing with the 3'-untranslated region causing translational repression and/or mRNA destabilization, which is sometimes through direct mRNA cleavage [21]–[23]. To date, thousands of miRNAs have been identified in a wide diversity of organisms including humans, leading to an actively expanding research field [24]. After over a decade of investigation of miRNAs, it is now clear that these non-coding RNA molecules serve a fundamental role in the regulation of gene expression; even though specific regulation and function of miRNAs is still largely unknown.

The expression profile and role of host miRNAs in the scenario of HIV-infection and AIDS progression has become a topic of interest. Several miRNAs have been described to interact either with the immune system related genes [25], [26] or the viral genes [27]–[29]. Despite recent studies have reported cellular miRNA profiles in several cohorts of HIV-infected patients [30]–[33], further studies are required in order to better understand the role of miRNAs in the field of HIV/AIDS. The assessment of how a specific miRNA profile could influence the different progression of HIV disease may be useful for understanding the basis of viral and immunological control for future functional therapeutic approaches. Thus, the aim of our study was to determine if there was a specific differential miRNA profile of Elite Controllers.

## Materials and Methods

### Study population

Samples were obtained from HIV-1-infected patients followed-up at the HIV Unit of the Hospital Clinic of Barcelona (Barcelona, Spain) between 1999 and 2009. Samples of non-infected donors, as a control group, were also obtained. The study was approved by the Institutional Ethics Committee and all participants gave written informed consent for miRNA profiling. Twenty-nine individuals, classified in 4 groups, were included in the study: HIV-negative individuals (HIV-; n = 5), Elite Controllers (EC; n = 8; viral load <50 cp/ml and CD4+ cell count >450 cells/mm^3^ for more than six years of follow-up in the absence of ART), Viremic Progressors (VP; n = 8; viral load >5000 cp/ml and CD4+ cell count >400 cells/mm^3^ for more than one year of follow-up in the absence of ART) and HIV-infected patients under antiretroviral treatment (ART; n = 8; viral load <50 cop/ml and CD4+ cell count >450 cells/mm^3^ for more than one year of follow-up). Medians were used to show central tendencies and interquartile ranges (IQR =  upper quartile Q3-lower quartile Q1) were calculated as measures of variability and statistical dispersion in each group.

### RNA isolation and quality control

Peripheral blood mononuclear cells (PBMCs) were either isolated from fresh blood by Ficoll-Hypaque gradient centrifugation or used after thawing. PBMCs (20×10^6^ cells) were cultured in RPMI medium containing 10% FBS and 2% gentamycin. Cells were stimulated with 1 ug/ml of phytohaemagglutinin (PHA; Sigma-Aldrich, St. Louis, Mo, USA) for 72 hours, washed in PBS (1×) and pelleted for RNA extraction. Total RNA (enriched for small RNA) was isolated according to manufacturer's instructions using the mirVana miRNA isolation Kit (Ambion, Huntingdon, UK). RNA concentration was calculated using NanoDrop technology ND-1000 (Thermo Scientific, Waltham, MA, USA). RNA integrity was then evaluated using RNA 6000 Nano LabChips on an Agilent 2100 Bioanalyzer (Agilent Technologies, Santa Clara, CA, USA). All chips were prepared according to the manufacturer's instructions at the Genomic platform of the CCiTUB (Centres Científics i Tecnològics University of Barcelona) located at the Barcelona Science Park (PCB). Total RNA degradation was evaluated by reviewing the electropherograms and the RNA integrity number (RIN) of each sample. Only samples with preserved 18S and 28S peaks and RIN values greater than 7 were selected for miRNA profile analysis.

### miRNA profiling using TaqMan low-density arrays (TLDA)

RNA (1 to 350 ng in 3 µl) was reverse transcribed using the miRNA reverse transcription kit in combination with the stem-loop Megaplex primer pool (Applied Biosystems, Foster City, CA, USA), allowing simultaneous reverse transcription of 381 small RNAs. miRNA expression profiles were acquired using TaqMan Array Human microRNA Card A v2.0 (Applied Biosystems, Foster City, CA, USA), containing 384 human miRNAs (hsa-miR). Reactions were performed using the Applied Biosystems 7900HT Fast Real-time PCR system. Reaction volumes contained: 50 µl of cDNA sample (30 to 1000 ng) in RNase-free water and 50 µl of (2×) TaqMan Universal PCR Master Mix. Thermocycler conditions were as follows: 94.5°C hot-start for 10 min, followed by 40 cycles of 97°C for 30 s and 59.7°C for 1 min.

### Accessibility of array data

Raw data and sets of filtered and global mean normalized data from TaqMan low-density arrays (TLDA) were deposited with the Gene Expression Omnibus (GEO, [34]) and are accessible at Series number GSE57323.

### TLDA data analysis

TLDAs were run in the in the 7900HT Fast Real-time PCR system using the SDS software v.2.3 (Applied Biosystems, Foster City, CA, USA) and raw Ct (cycle threshold: the number of cycles required for the fluorescent signal to cross the threshold) values of the expression of each individual miRNA were obtained using automatic thresholding of all the processed data together with the StatMiner Software (Integromics, Granada, Spain). Those miRNAs with Ct values >35 and not amplified wells were omitted from the analysis. Moreover, miRNAs that were not expressed in more than 25% of the samples, belonging to each group of study, were also excluded from the analysis. For each individual sample, global mean normalization strategy [35], [36] was performed calculating the ΔCt values for each miRNA (ΔCt  =  Ct_target miRNA_ – mean Ct_all assessed miRNAs_). A non-parametric Mann-Whitney U test was run in MEV software V4.5 [37] for statistical comparisons between group-pairs. Benjamini-Hochberg correction test was applied as an estimated false discovery rate (FDR) of 5%, controlling for the expected proportion of incorrectly rejected null hypotheses [38]. Samples were clustered, comparing EC and VP, by their miRNA expression profiles using the hierarchical clustering algorithm of the software. The Euclidean distance-metric hierarchical cluster represented up-expressed miRNAs in red tones and down-expressed miRNAs in green tones. Fold change (log_2_) expression of differentially expressed individual miRNAs in EC and VP relative to HIV- and ART were calculated as 2^-ΔΔCt^ (ΔΔCt  =  ΔCt_EC or VP_ – ΔCt_HIV- or ART_). A fold change value closer to “0” would indicate a similar expression level compared to reference group, whereas a positive/negative value would represent an up/down-expressed level. Two-way analysis of variance (ANOVA) tests were performed for global comparisons and Bonferroni post-tests for replicate-means comparisons using GraphPad Prism 5.0.

### Validation of results

Those differentially expressed miRNAs with a significance p-value ≤0.001 were re-assessed through individual RT-qPCR assay (Applied Biosystems, Foster City, CA, USA). Furthermore, in order to strengthen the observed expression profiles, a validation cohort consisting of 8 HIV-, 13 EC, 14 VP and 14 ART new patients was added to the study. Individual RT-qPCR assays of the differentially expressed miRNAs of interest were performed in this validation cohort.

RNA (10 ng) was reverse transcribed in 15 µl according to manufacturer's recommendations using TaqMan miRNA reverse transcription kit (Applied Biosystems, Foster City, CA, USA). miRNA expression assays were carried out using TaqMan primers and probes (Applied Biosystems, Foster City, CA, USA) for endogenous control small RNAs RNU44 (ID 001094) and RNU48 (ID 001006) and target miRNAs. Relative quantifications (RQ) were performed using the Applied Biosystems 7900HT Fast Real-time PCR system. Reaction volumes contained: 7.67 µl of water, 1 µl of TaqMan primer/probe mix for target or endogenous control small RNA, 10 µl of (2×) Universal master mix (Applied Biosystems, Foster City, CA, USA) and 1.33 µl of cDNA at a final concentration of 10 ng. Thermocycler conditions were as follows: 95°C hot-start for 10 min, followed by 40 cycles of 95°C for 15 s and 60°C for 1 min. Raw Ct values were exported from the SDS software v.2.3 to the RQ Manager v1.2 softwear (Applied Biosystems, Foster City, CA, USA) for ΔCt (ΔCt  =  Ct_target miRNA_ – mean Ct_endogenous small RNAs_) value determination as the normalization method. Fold change (log_2_) expression levels of the individual miRNAs in EC, VP, ART, relative to HIV-, were calculated as 2^−ΔΔCt^ (ΔΔCt  =  ΔCt_EC, VP, ART_ – ΔCt_HIV-_). One-way analysis of variance (ANOVA) tests were performed for global comparisons and Turkey post-hoc tests for pair comparisons using GraphPad Prism 5.0 (GraphPad Software, La Jolla, CA, USA).

## Results

### Characteristics of the study participants

Characteristics of the study participants of the screening and the validation cohorts are shown in Table 1. None of them was co-infected by hepatitis C virus (HCV). After seven years of follow-up all the participants from the EC group maintained viral load <50 cp/ml and CD4+ cell count >450 cells/mm^3^. A heterogeneous distribution of HLA-B57*01 was observed and none of them showed the *CCR5* delta-32 gene deletion (data not shown). No statistically significant differences were observed in any comparison except in the time since HIV diagnosis (p = 0.002) and time of exposure to antiretroviral therapy (p = 0.002) between the ART groups of the screening and the validation cohorts.

**Table 1:** Baseline characteristics of the study participants.

	Screening Cohort				Validation Cohort			
Participant characteristics	HIV- (n = 5)	VP (n = 8)	EC (n = 8)	ART (n = 8)	HIV- (n = 8)	VP (n = 14)	EC (n = 13)	ART (n = 14)
Age, years*	34 (9)	42.5 (8)	48 (28)	46 (2)	32 (7)	38 (7.75)	49 (20.5)	47 (18.75)
Men/women, n men (%)	2 (40)	7 (87.5)	7 (87.5)	6 (75)	3 (37.5)	13 (92.9)	8 (61.5)	12 (85.7)
Presumed mode of HIV transmission, n (%)								
MSM	N/A	6 (75)	5 (71.5)	3 (37.5)	N/A	12 (85.7)	5 (38.5)	9 (64.3)
heterosexual	N/A	2 (2)	0 (0)	4 (50)	N/A	1 (7.15)	3 (23.1)	2 (14.3)
Other/Unknwon	N/A	-	2 (28.5)	1 (12.5)	N/A	1 (7.15)	5 (38.5)	3 (21.5)
Time since HIV diagnosis, years*	N/A	9 (3.75)	13 (8)	16 (3)	N/A	4.5 (5.25)	10 (8)	9 (9.5)
HIV viral load (log)*	N/A	4.6 (5.12)	1.6 (0.27)	1.56 (0.09)	N/A	4.27 (0.35)	1.57 (0.01)	1.57 (0.01)
CD4+ T-cell count (cells/µl)*	N/A	606 (281)	687 (297)	962.5 (887)	N/A	626.5 (158)	715 (597)	772.5 (354)
Nadir CD4+ T-cell count (cells/µl)*	N/A	466 (88)	600 (332)	348.5 (288)	N/A	445.5 (238)	523.5 (296)	342.5 (193)
Time of exposure to ART, years*	N/A	N/A	N/A	15.5 (1)	N/A	N/A	N/A	6 (10.25)

VP, viremic progressors; EC, elite controller; ART, antiretroviral therapy; MSM, men who had sex with men; *; median [interquartile range  =  upper quartile (Q3) - lower quartile (Q1)]; N/A, not applicable.

### Taqman Low-density miRNA arrays reveal differentially expressed miRNAs

All RNAs were suitable (RIN >7) for the miRNA expression profile analysis through the TaqMan Array Human microRNA Cards v3.0. Expression profiles of HIV-, EC, VP, and ART individuals were acquired after adequate normalisation steps for statistical analysis. Finally, 286 (38%) miRNAs were included in the analysis once the exclusion criteria were carried out. Mann-Whitney U test set to a false discovery rate (FDR) of 5% provided a set of miRNAs differentially expressed in each group-pair: 52 miRNAs in HIV- vs ART, 23 miRNAs in EC vs VP, 22 miRNAs in EC vs ART and 25 miRNAs in VP vs HIV-. No differential miRNAs were observed when comparing neither EC vs HIV- nor VP vs ART; EC were statistically similar to HIV- and VP to ART in terms of PHA-activated PBMC miRNA profile (Table S1).

### Hierarchical clustering: miRNA profile in Elite Controllers differs from Viremic Progressors

**Figure 1:**
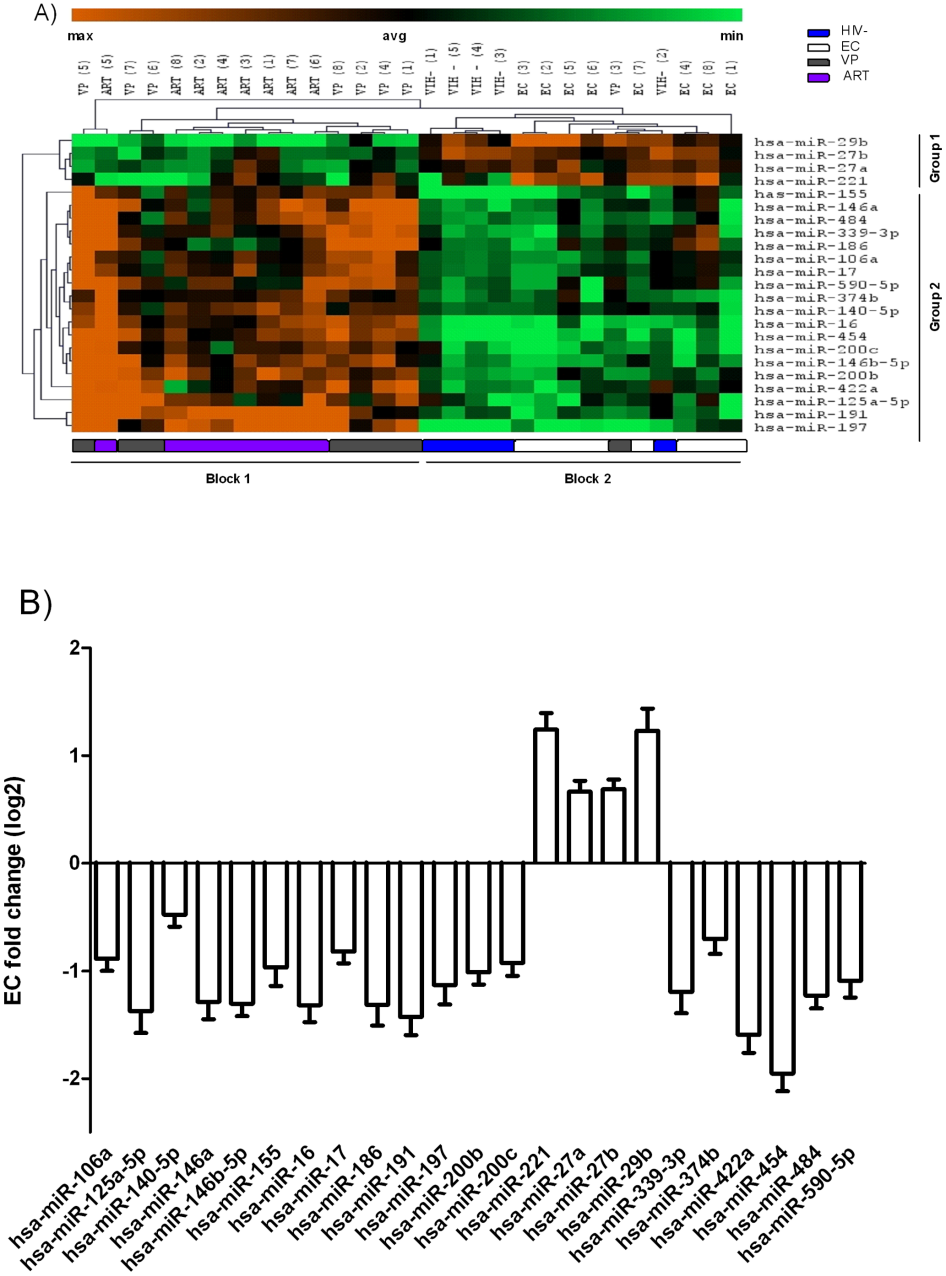
Differential miRNAs between Elite Controllers (EC) and Viremic Progressors (VP). A) Hierarchical clustering of the differentially expressed miRNAs between EC and VP. Patients are ordered on vertical lines and candidate miRNAs on horizontal lines. For each miRNA, green represents under-expressed and red over-expressed values compared to the average value (median), in dark. Dendrograms (tree graph) between patients and between miRNAs are depicted, where the closest branches of the tree represent patients/miRNAs with the most similar expression pattern. Two blocks of patients (Block 1/Block 2) with an inverse expression profile were segregated. Two groups of miRNAs (Group 1/Group 2) with an inverse expression profile were segregated within each block. B) Fold change (log 2) of the 23 differentially expressed miRNAs in EC. Differential levels are normalized to all assesed miRNAs and relative to VP. Bars represent standard error means (SEM).

Twenty-three differentially expressed miRNAs resulting from the Mann–Whitney U test (5% FDR) (Table S1) were classified by hierarchical clustering (average linkage clustering constructed on Euclidian distances) (Figure 1A). The analysis segregated the population in two separate blocks (block1/block2). Block 1 included EC and HIV- with no significant differences on miRNA expression. Viremic progressor patient number 3 clustered together within this block, making this block slightly heterogeneous. Block 2 clustered VP and ART patients. A set of 4 miRNAs with an inverse expression profile between the two blocks, subdivided each block of patients into two groups of miRNA (group1/group2). On the one hand, these 4 miRNAs (hsa-miR-221, hsa-miR-27a, hsa-miR-27b and hsa-miR-29b) were down-expressed in VP and ART (block1-group1) and up-expressed in EC and HIV- (block2-group1). On the other hand, these 19 miRNAs (hsa-miR106a, hsa-miR-125a, hsa-miR-140, hsa-miR-146a, hsa-miR-146b, hsa-miR-155, hsa-miR-16, hsa-miR-17, hsa-miR186, hsa-miR-191, hsa-miR-197, hsa-miR-200b, hsa-miR-200c, hsa-miR-339, hsa-miR-374, hsa-miR-422, hsa-miR-422, hsa-miR-454, hsa-miR-484 and hsa-miR-590) were up-expressed in VP and ART (block1-group2) and down-expressed in EC and HIV- (block2-group2). Expression of the differentially expressed miRNAs in EC was measured as fold change (log_2_) relative to VP: hsa-miR-221 and hsa-miR-29b were the most highly expressed miRNAs (fold change 1.24 and 1.23, respectively) and hsa-miR-454 showed the lowest expression (fold change -1.95) in EC when compared to VP (Figure 1B).

#### miRNA profile in Elite Controllers differs from Viremic progressors and treated individuals

**Figure 2:**
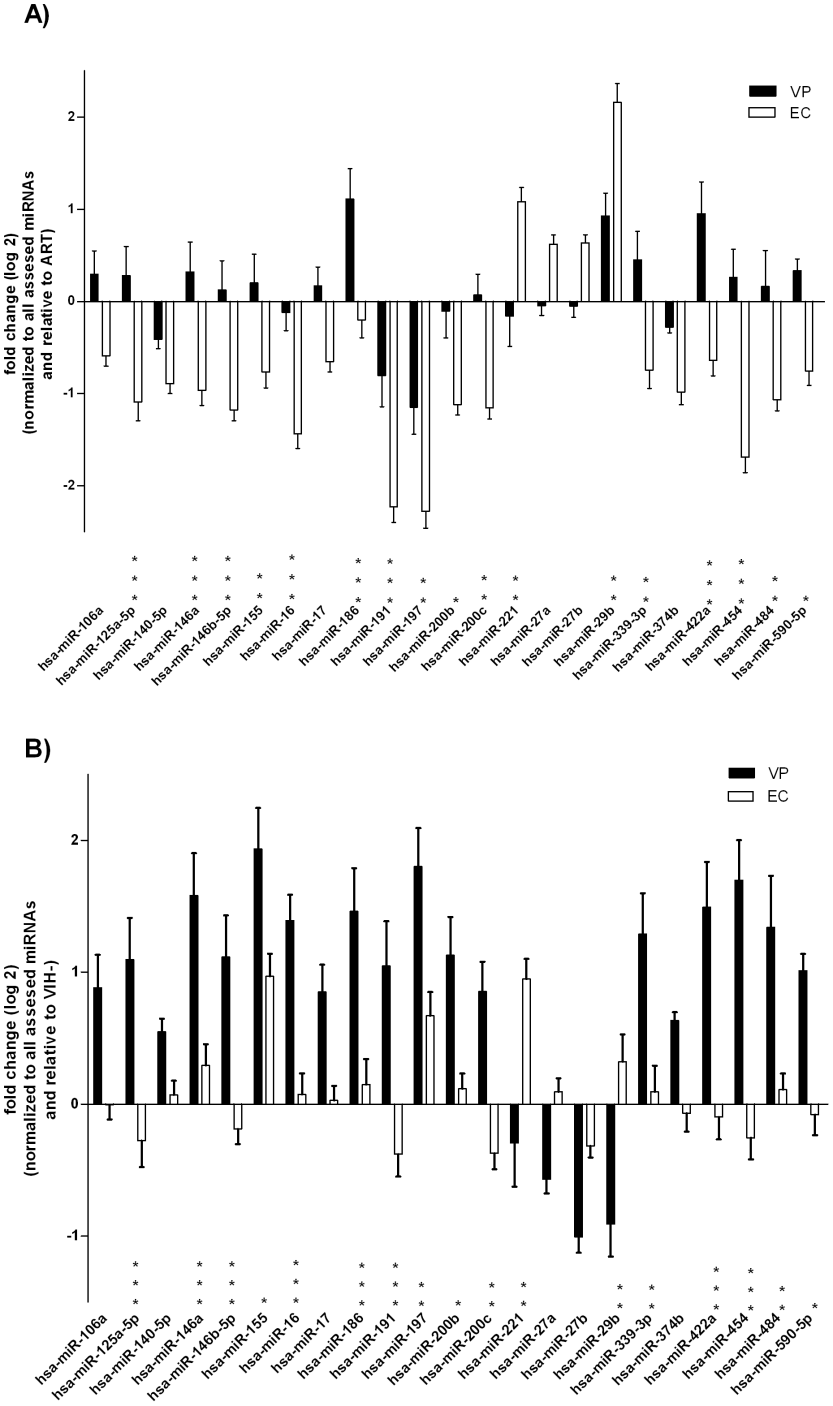
Fold change (log_2_) of the 23 differentially expressed miRNAs in EC and VP. A) normalized to all assessed miRNAs and relative to ART, B) normalized to all assessed miRNAs and relative to HIV-. Bars represent standard error means (SEM); *, p<0.05; **, p<0.01; ***, p<0.001; VP, viremic progressors; EC, elite controllers; ART, antiretroviral therapy.

Differential miRNA expression between EC and VP was measured as fold change (log_2_) relative to ART (Figure 2A). Overall, EC showed a down-expressed miRNA profile compared to the ART group except for hsa-miR-221, -27a, -27b, -29b levels. hsa-miR-29b was the most highly expressed miRNA (fold change 2.1) and hsa-miR-197 the one with the lowest expression level (fold change -2.2) compared to ART. Six miRNAs were statistically similar (p<0.05) between EC and VP when compared to ART: hsa-miR-106a, -140-5p, -17, -27a, -27b and -374b. In summary, VP showed a closer profile (global mean fold change of 0.1) to the ART group than the EC (global mean fold change of -0.69) (Table S2 A).

#### miRNA profile in Elite Controllers is similar to non-infected individuals

Differential miRNA expression between EC and VP was measured as fold change (log_2_) relative to HIV- (Figure 2B). Hsa-miR-221 and hsa-miR-155 were the most highly expressed miRNAs (fold-change 0.9) and hsa-miR-191 and hsa-miR-200c were the ones with the lowest expression levels (fold change -0.3) compared to HIV-. Interestingly, has-miR-155 was the most up-expressed miRNA in both groups compared to HIV- being the expression level significantly higher (p<0.05) in VP. Six miRNAs were statistically similar (p<0.05) between EC and VP compared to HIV-: hsa-miR-106a, -140-5p, -17, -27a, -27b and -374b. In summary, EC showed a closer profile (global mean fold change of 0.08) to the ART group than the VP (global mean fold change of 0.87) (Table S2 B).

### Validation of expression profiles through individual RT-qPCR assays

Differentially expressed miRNAs between EC and VP, with a significance p-value ≤0.001 (n = 5, 22%), were successfully validated through individual RT-qPCR assays in the same study population (data not shown).

Moreover, in order to strengthen the tendencies observed in the miRNA profiling analysis, four miRNAs of interest were re-assessed in a validation cohort with similar characteristics to the screening cohort (Table 1).

**Figure 3:**
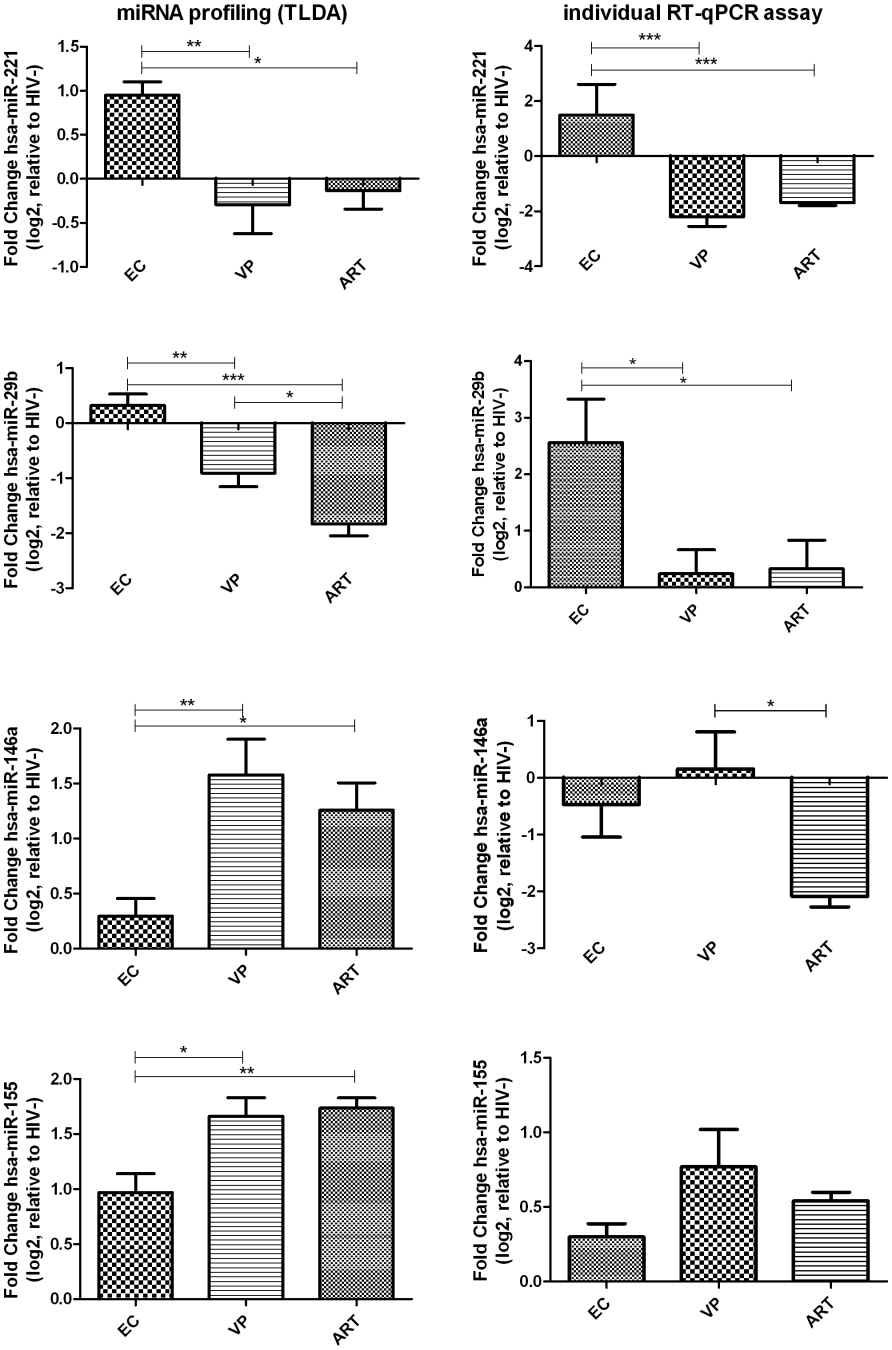
Fold change (log_2_) of EC, VP and ART, normalized to endogenous control miRNAs and relative to HIV-. One-way analysis of variance (ANOVA) tests were performed for global comparisons and Turkey post-hoc tests for pair comparisons. Bars represent standard error means (SEM); *, p<0.05; **, p<0.01; ***, p<0.001 for Tuckey post-hoc tests; VP, viremic progressors; EC, elite controllers; ART, antiretroviral therapy.

Validation cohort was analysed for the expression levels of hsa-miR-221, -29b, -146a and -155 between EC, VP and ART, relative to HIV- (Figure 3). Although significant differences were only observed for hsa-miR-221 (p<0.001) and hsa-miR-29b (p<0.05), the four miRNAs of interest reflected the same expression tendencies observed in the profiling analysis: EC showed up-expressed levels of hsa-miR-221 and hsa-miR-29b and lower levels of hsa-miR146a and hsa-miR-155 compared to VP. However, individual RT-qPCR assay for hsa-miR-146a did not reproduce the expression levels observed in the profiling analysis in any of the three groups of study. Moreover, the ART group from the validation cohort did not imitate the expression levels of hsa-miR-29b and hsa-miR-146a shown in the profiling analysis.

## Discussion

The goal of our study was to assess potential miRNAs that are differentially expressed in HIV-1-infected patients who control viremia in the absence of antiretroviral therapy: Elite Controllers. For that purpose, miRNA profile of 29 individuals categorized in EC, VP, ART and HIV-, was obtained from PHA-activated PBMCs. Even though the heterogeneity observed within EC in previous transcriptome studies [17], [39], our results show a specific differential miRNA pattern in EC when compared to VP. Our findings revealed 23 differentially expressed miRNAs in EC that are present in similar levels in HIV- but dissimilarly in VP and ART. In order to validate the expression levels observed, those miRNAs with a significance p-value ≤0.001 between EC and VP were validated through individual RT-qPCR assays. Our results are consistent with studies reporting that either PBMCs or specific blood cell population miRNA profile of HIV-infected elite controllers, resembles that of HIV- individuals [30], [40]. A recent study comparing PBMC miRNA profiles between HIV-infected individuals with low or undetectable viral load and uninfected subjects, conclude that similar patterns are observed across the study groups [32]. However, the suppressed patients used in this last study were all on antiretroviral therapy at the moment of sample collection, a fact that suggest HIV-1 to be able to induce a miRNA dysregulation. Indeed, previously published data showed a major down-regulation of most of the miRNAs in HIV-infected patients [30], [31] whereas in the current study, where stimulated PBMCs were used, we observed a trend to a preferential miRNA down-regulation in EC and non-infected subjects as well as a major up-regulation of the differential miRNAs in non-suppressed HIV-positive individuals, probably as a consequence of the effects of the in vitro T-cell stimulation.

On the one hand, EC showed up-expressed levels of hsa-miR-221, hsa-miR-27a, hsa-miR-27b and hsa-miR-29b compared to VP. These miRNAs are highly expressed in PBMCs [41] and are plausible molecular candidates to be involved in HIV replication and infectivity. Human miR-29b has been previously described as one of the profile components of EC [30] and to be related with infected patients with low viral load [31]. Additionally, previous data report the implication of hsa-miR-29b in HIV replication through targeting the virus in the transcribed 3′-LTR region [42] or regulating the viral negative regulatory factor (nef) [43]. Nef highjacks MHC (mayor histocompatibility complex)-class I along with other molecules impeding a correct antigen presentation [44], [45]. Moreover, hsa-miR-29b and hsa-miR-27b have been described to repress the translation of cellular cofactor cyclin T1 in resting and activated CD4+ T-cells. Cyclin T1 binds the viral trans-activator of transcription (tat) and activates the transcription of the integrated provirus [46]. Cellular levels of integrated viral DNA have been described to be much lower in elite controllers compared to other patients on and off anti-retroviral drugs [47]. VP and ART patients might express these miRNAs in lower levels in order to avoid cyclin t1 suppression and allow replication of integrated provirus. A second miRNA, newly identified in this work, to be potentially related to the control of viral infectivity is hsa-miR-221. In this case, previous data reports a functionality in the control of Intracellular Adhesion Molecule-1 (ICAM-1) expression levels either through the IFN-alpha pathway or by direct targeting [48], [49]. Cellular levels of ICAM-1 influence HIV-1 infectivity and viral dissemination [49]-[52]. Considering the mentioned functional analyses, up-expression of these two molecules in EC could suggest an improved viral control and antigen presentation through miR-29b and a reduced viral infectivity through miR-221, although this should be more accurately investigated.

On the other hand, 19 miRNAs were significantly down-expressed in EC. Out of these miRNAs, hsa-miR-146a and hsa-miR-155 became of our interest due their important role in a wide spectrum of immune compartments. Both miRNAs were up-expressed in VP and this pattern has already been correlated with high viral load [31], [32]. The co-activation of hsa-miR-146 and hsa-miR-155 results in a transcriptional activation of NF-kB target genes that encode various mediators of inflammation, such as cytokines, acute phase proteins and inducible enzymes against a variety of microbial components [53]. Subsequent findings showed that both hsa-miR-146 and -155 targeted mRNAs in the signalling cascade of toll-like receptor 4 (TLR4) and bolstered the link with NFkB-regulated innate immunity, leading to a model in which these two miRNAs facilitate a negative-feedback loop that may protect from an excessive TLR4 response [54]. Other groups have recently found that hsa-miR-155 was strongly expressed in effector/memory Tregs [55]. Levels of effector/memory Tregs are significantly increased in different HIV progression profiles (HIV-infected individuals with progressive infection versus long term non-progressors). Thus, we stress the importance of analysing the expression of these molecules in different T-cell subsets to better understand its role in HIV pathogenesis.

Moreover, hsa-miR-155 has been shown to be involved in the differentiation from naive to effector CD8+ T cells being required for effective CD8+ T cell responses to virus infections through modulation of responsiveness to type I interferon [56]-[58]. Down-expression of these two molecules in EC would suggest less inflammatory status, a minor activation of the immune system and a better antiviral immune response. In fact, previously data suggested the contribution of miR-155 to the pathogenesis of HIV-1 infection in HIV naïve individuals [29].

In order to give consistency to the tendencies of the miRNA profiles observed, a validation cohort was analysed through individual RT-qPCR assay for four differentially expressed miRNAs of interest: hsa-221, -29b, -146a, -155. Re-assessment of these miRNAs in a new set of patients reflected the same tendencies observed in the profiling analysis between EC and VP, although significant differences were only observed for hsa-miR-221 (p<0.0001) and hsa-miR-29b (p<0.05). The ART group from the validation cohort did not imitate the expression levels of hsa-miR-29b and hsa-miR-146a shown in the profiling analysis. Intriguingly, ART patients from the two cohorts (screening and validation) differed in the time of exposure to ART [median (IQR)]: 15.1 (1) years in the screening cohort and 6 (10.25) years in the validation cohort and in the time since HIV diagnosis [median (IQR)]: 16 (3) years in the screening cohort and 9 (9.5) years in the validation cohort. This observation leads us to a new hypothesis that questions whether these variables might influence miRNA levels.

The findings described herein should be considered with caution due to the limitations of our study. First of all, although the screening results have been validated by enlarging the number of individuals per group, we are conscious of the degree of variation that could occur by the limited sample size used. Moreover, our experimental design does not allow us to attribute the different pattern of miRNA found to any particular specific cell subset. Lastly, no functional data focused on the differential miRNAs is described in the current manuscript. In order to shed more light to all these questions, new experiments should be performed in the future.

In summary, our study reveals a differentially expressed miRNA profile in Elite Controllers that is similar to non-infected individuals and differs from Viremic Progressors who are closer to treated individuals. Some of these differential miRNAs have been reported to be involved in the control of viral replication, viral infectivity, immune activation, and modulation of both innate and acquired immune responses. Nevertheless, more studies are needed in order to dissect the relevant roles of miRNAs in various states of HIV infection and its use as a potential prognostic marker in disease progression or as a future therapeutic approach.

## Supporting Information

Table S1 **Statistically significant differential miRNAs between analysed group-pairs.**
(DOCX)

Table S2 **Fold change (log2) of differentially expressed miRNAs in Elite Controllers (EC) and Viremic progressors (VP) normalized to all assessed miRNAs and relative to A) VIH- and B) patients under ART.**
(DOCX)
