# Supplementary material for: Differential MicroRNA Expression Profile between Stimulated PBMCs from HIV-1 Infected Elite Controllers and Viremic Progressors
Source: PLoS One. 2014 Sep 16;9(9):e106360. doi: 10.1371/journal.pone.0106360 (PMC4165582; doi:10.1371/journal.pone.0106360)
Supplement: Table S1 — Statistically significant differential miRNAs between analysed group-pairs. (DOCX) [file pone.0106360.s001.docx]

Suplementary_Table S1. Statistically significant differential miRNAs between analysed group-pairs.

*HIV-, unifected individual;, ART, antiretroviral treatment; EC, elite controllers; VP, viremic progressors; B-H, Benjamini-Hochberg correction through 5% FDR; na, not applicable.*
