# Supplementary material for: Differential MicroRNA Expression Profile between Stimulated PBMCs from HIV-1 Infected Elite Controllers and Viremic Progressors
Source: PLoS One. 2014 Sep 16;9(9):e106360. doi: 10.1371/journal.pone.0106360 (PMC4165582; doi:10.1371/journal.pone.0106360)
Supplement: Table S2 — Fold change (log2) of differentially expressed miRNAs in Elite Controllers (EC) and Viremic progressors (VP) normalized to all assessed miRNAs and relative to A) VIH- and B) patients under ART. (DOCX) [file pone.0106360.s002.docx]

Supplementary Table S2. Fold change (log2) of differentially expressed miRNAs in Elite Controllers (EC) and Viremic progressors (VP) normalized to all assessed miRNAs and relative to A) VIH- and B) patients under ART. SEM, standard error mean; p, p-value.

*HIV-, unifected individual;, ART, antiretroviral treatment; EC, elite controllers; VP, viremic progressors.*
